# Vitamin C in Cancer: A Metabolomics Perspective

**DOI:** 10.3389/fphys.2018.00762

**Published:** 2018-06-19

**Authors:** Seyeon Park, Seunghyun Ahn, Yujeong Shin, Yoonjung Yang, Chang H. Yeom

**Affiliations:** ^1^Department of Applied Chemistry, Dongduk Women’s University, Seoul, South Korea; ^2^Department of Food and Nutrition, Dongduk Women’s University, Seoul, South Korea; ^3^YCH Hospital, Seoul, South Korea

**Keywords:** vitamin C, cancer, metabolomics, glutathione metabolism, glucose metabolism

## Abstract

There is an ongoing interest in cellular antioxidants and oxidants as well as cellular mechanisms underlying their effects. Several reports suggest that vitamin C (L-ascorbic acid) functions as a pro-oxidant with selective toxicity against specific types of tumor cells. In addition, reduced glutathione plays an emerging role in reducing oxidative stress due to xenobiotic toxins such as metals and oxidants associated with diseases such as cancer, cardiovascular disease, and stroke. High-dose intravenous vitamin C and intravenous glutathione have been used as complementary, alternative, and adjuvant medicines. Here, we review the molecular mechanisms underlying the regulation of oxidation/reduction systems, focusing on the altered metabolomics profile in cancer cells following treatment with pharmacological vitamin C. This review focuses on the role of vitamin C in energy metabolism in terms of adenosine triphosphate, cysteine, and reduced glutathione levels, affecting cancer cell death.

## Systems Biology Perspectives on Vitamin C

From a systems biology perspective, the integrated use of proteomics, genomics, and transcriptomics is extremely important for translational metabolomics-based research (Shin et al., 2016). Microarray analysis and qPCR have been performed to investigate the effect of vitamin C on gene expression. A recent study has reported that a series of genes in embryonic stem cells are differentially regulated by vitamin C treatment (Shin et al., 2004). Most of these upregulated genes belong to gene families that regulate neurogenesis, neuronal maturation, and neurotransmission (Shin et al., 2004; Belin et al., 2010). Based on the observation that vitamin C treatment suppresses the expression of *PMP22*, a myelin gene that is overexpressed in one of the hereditary motor and sensory neuropathies, it has been suggested that vitamin C induces dose-dependent suppression of *PMP22* expression by inhibiting the production of cAMP, a regulator of CREB-binding promoter located in *PMP22* (Hai et al., 2001; Kaya et al., 2007; Belin et al., 2010). Vitamin C acts as a competitive inhibitor of adenylate cyclase, and represses the expression of a variety of genes under the control of cAMP-dependent pathway (Belin et al., 2010). Microarray data suggested that 5 days of vitamin C supplementation under normal physiological condition, but not under cancer condition, induce an upregulation of calnexin isoform (Canali et al., 2014). On the other hand, microarray analysis using human colon carcinoma HT29 cells has shown that vitamin C downregulated the expression of translational initiation factor subunits, tRNA synthetases, and genes crucial for cell cycle progression accompanied by S-phase arrest of proliferative cells induced by vitamin C (Belin et al., 2009). In addition, microarray analysis using mouse models grafted with HT29 cells has consistently shown a decreased expression of translational initiation factor and tRNA synthetases in tumors following vitamin C treatment (Belin et al., 2009).

Proteomics research also elucidates the protein expression in terms of post-translational modifications triggered by a specific stimulus independent of protein neo-synthesis. Post-translational modifications such as phosphorylation of tyrosine or serine/threonine, sulfur oxidation of cysteine, and glutathionylation represent key mechanisms of cell stimulation related to oxidative stress. Our group conducted proteomics analyses of the effect of vitamin C on cancer at the cellular level and in mouse models grafted with tumor cells (Park et al., 2006, 2009). When human leukemia cell line NB4 was treated with relatively high concentration (0.5 mM) of vitamin C, approximately 200 differentially expressed spots were detected by two-dimensional electrophoresis. This proteomics analysis suggested that the domain polymerization state of quaternary structure protein composed of four domains via disulfide bond was altered in response to vitamin C treatment. One of these proteins included protein disulfide isomerase (PDI) belonging to the thiol/disulfide exchange catalyst superfamily. It acts as a protein-thiol-oxidoreductase enzyme. It also shares sequence homology with thioredoxin (Park, 2013). Another protein was immunoglobulin heavy chain binding protein (BiP), a multi-domain chaperone identical to chaperone Hsp70. BiP binds via a disulfide bond to the α-subunit of prolyl 4-hydroxylase (P4-H), a partner of PDI (John and Bulleid, 1996). P4-H is a multimeric protein composed of α-subunit and β-subunit. Its α-subunit is catalytically more important than its β-subunit. In addition, its β-subunit is identical to the multifunctional PDI enzyme (John and Bulleid, 1996). These results suggest that vitamin C oxidizes intracellular levels of reduced glutathione and the valence change of glutathione and reduced glutathione results in disulfide bond rearrangement in the quaternary structure of proteins such as PDI and BiP. Our previous study also demonstrated that changes in intracellular valence of glutathione between reduced glutathione occur shortly after exposure to vitamin C (Park et al., 2004).

Regional changes in oxidation state induced by vitamin C lead to a variety of alterations involving sulfur oxidation in the cellular milieu and result in transitions in the protein quaternary structure. The oxidation state of cysteine sulfur is important for the determination of the tertiary structure of proteins (Park, 2013). An important example of protein influenced by regional changes in oxidative state associated with vitamin C is glyceraldehyde 3-phosphate dehydrogenase (GAPDH) involved in glycolysis metabolism. It has been reported that GAPDH activity is reduced by reactive oxygen species (ROS) or vitamin C treatment (Hwang et al., 2009; Yun et al., 2015). High concentration of vitamin C generating ROS suppresses GAPDH via Cys glutathionylation (Hwang et al., 2009; Yun et al., 2015). The role of GAPDH in vitamin C-dependent alterations suggests that vitamin C influences glucose metabolism via altered oxidation/reduction status. It also suggests an interface between proteomics analysis and metabolomics approach to determine the effect of vitamin C.

## Metabolomics Overview

Metabolomics is appropriate for the study of biological processes induced by endogenous developmental changes or drugs and other xenobiotics via endogenous metabolome (Oskouie and Taheri, 2015). Approximately 38000 chemical compounds in metabolites are generally detected in the human body according to a recent report (Oskouie and Taheri, 2015). Metabolome is typically composed of carbohydrates, amino acids, lipids, nucleotides, and other organic compounds. Metabolites exhibit varying levels of volatility and polarity, and therefore, a variety of analytical technologies are employed in metabolomics studies (Oskouie and Taheri, 2015). The most common methodologies used for the identification of metabolites include nuclear magnetic resonance (NMR) spectroscopy and mass spectrometry (Meister and Anderson, 1983).

## Vitamin C Affects Cancer via Glucose Metabolism

### Glucose Metabolism in Cancer Cell

Metabolic profiling indicates altered metabolomics in the cancer cells during cancer progression. Therefore, metabolite-based investigations of various cancers represent a useful approach to identify diagnostic, therapeutic, and prognostic biomarkers in cancer. It has been suggested that glucose metabolism in tumor cells varies from that of normal cells. Glucose metabolism is known to be associated with sustainable proliferation. Otto Warburg suggested that tumor cells metabolize approximately ten-fold more glucose to lactate under aerobic conditions than normal tissues in a given time (Koppenol et al., 2011). Moreover, higher conversion of glucose to lactic acid under aerobic condition in cancer cells is accompanied by retaining mitochondrial respiration (Koppenol et al., 2011).

Specific metabolic proteins have been identified as potential oncoproteins (**Table 1**). For example, pyruvate kinase type M2 is an oncoprotein expressed in squamous cell carcinoma (Wong et al., 2008). Specific oncoproteins alter cancer cell metabolism by directly regulating key metabolic enzymes and pathways (Nagarajan et al., 2016). For example, oncogenic transcription factor MYC activates the transcription of glycolytic enzyme genes and glucose transporters that enhance aerobic glycolysis (Shim et al., 1997; Osthus et al., 2000; Ahuja et al., 2010). In addition, oncogenic kinase Akt activates hexokinase 2, phosphofructokinase 1 (PFK1), and phosphofructokinase 2 (PFK2). It also induces localization of glucose transporters to the cell surface, resulting in enhanced glycolysis (Deprez et al., 1997; Robey and Hay, 2009). It is well known that mitochondrial metabolism is regulated by oncoprotein Bcl-2 (Krishna et al., 2011). Ha-Ras and β-catenin oncoproteins reprogram metabolic flows in mouse liver tumors (Unterberger et al., 2014). Hepatitis B X-interacting protein, an oncoprotein, also enhanced glucose metabolism by suppressing the synthesis of cytochrome c oxidase 2 and pyruvate dehydrogenase alpha 1 in breast cancer (Liu et al., 2015).

**Table 1:** Metabolic proteins identified as oncoproteins.

Metabolic oncoproteins	Regulation	Cancer type (Reference)
Pyruvate kinase type M2	Enhance glycolysis	Squamous cell carcinoma (Wong et al., 2008).
MYC	Enhance glycolysis (activates the transcription of glycolytic enzyme genes and glucose transporters)	Lymphoma cell lines (Shim et al., 1997). Myc-transformed fibroblast cells (Osthus et al., 2000). Mice myocardium (Ahuja et al., 2010)
Akt	Enhance glycolysis (activates hexokinase 2, PFK1, and PFK2)	Mammalian cells with hyperactive Akt (Robey and Hay, 2009)
Bcl-2	Enhance oxidative phosphorylation	Human lymphoma (Krishna et al., 2011)
Ha-Ras	Enhance glycolysis	Ha-Ras-mutated mouse model (Unterberger et al., 2014)
β-Catenin	Enhance glycolysis	Ha-Ras-mutated mouse model (Unterberger et al., 2014)
Hepatitis B X-interacting protein	Enhance glycolysis	MCF7 (Liu et al., 2015)

Parkin (PARK2), a Parkinson disease-associated gene, is a glucose metabolism-related tumor suppressor whose expression is diminished in tumors (Zhang et al., 2011). Parkin deficiency activates glycolysis and reduces mitochondrial respiration, leading to the Warburg effect (Zhang et al., 2011). In Parkinson deficient cells, mitochondrial dysfunction and enhanced oxidative stress are observed (Zhang et al., 2011).

Other evidence implicating oncogenes in aerobic glycolysis include phosphorylation of a variety of glycolytic enzymes by oncogenic Src kinase and enhanced glucose uptake by oncogenic Ras activation in fibroblasts (Cooper et al., 1984; Flier et al., 1987). Ras oncogene links metabolomic alterations by vitamin C with tumor suppression by vitamin C. A recent report found that high-dose vitamin C is selectively toxic to human colorectal cancer cells carrying either K-Ras or B-Raf mutations (Yun et al., 2015). Mutant K-Ras or B-Raf activate downstream mitogen-activated protein kinase (MAPK) pathway, leading to the up-regulated expression of GLUT1, a glucose transporter that imports dehydroascorbate (DHA, an oxidized form of vitamin C) into cells (Yun et al., 2009, 2015). Imported DHA is then reduced back to vitamin C by oxidizing glutathione, resulting in depletion of glutathione, and high levels of intracellular ROS (Vera et al., 1993, 1995; Yun et al., 2015). It has been suggested that such oxidative stress in highly glycolytic K-Ras- or B-Raf-mutant cells triggers inactivation of GAPDH via Cys oxidation, leading to abnormal glycolysis that is rarely seen in K-Ras or B-Raf wild-type cells (Yun et al., 2015).

In addition to glucose metabolism, vitamin C induces specific changes in other cellular metabolic pathways in cancer cells. Oxidative stress is an important mechanism of vitamin C in cancer cells. Glutathione-related metabolism also affects cancer progression by vitamin C because glutathione is a major cellular antioxidant.

## Redox Metabolism via Glutathione in Cancer Cells

An underlying hypothesis is that ROS production is an inevitable consequence of electron transport combined with oxidative phosphorylation under physiological conditions. High levels of ROS induce cellular senescence or death. However, oxidation-evading mechanisms of tumor cells differ from that of normal cells (Andrisic et al., 2018). As discussed above, a distinct feature of many cancer cells is their metabolic dependence on anaerobic glycolysis in spite of functional glucose metabolism at the expense of oxygen (Koppenol et al., 2011). Although energetically less efficient, glycolysis produces ATP at a much faster rate by avoiding mitochondrial oxidative phosphorylation (Andrisic et al., 2018). Therefore, cancer cells are protected from deleterious ROS generation that normally should be expected during enhanced proliferation (Andrisic et al., 2018). In addition, enhanced glycolysis is likely to act as a pentose phosphate pathway shunt to provide NADPH and substrates for nucleotide synthesis. NADPH also acts as a reducing agent for oxidized GSH and provides intracellular redox balance (Andrisic et al., 2018). Nonetheless, production of ROS is stimulated in cancer cells compared with that of normal cells (Trachootham et al., 2009). Therefore, cancer cells generally up-regulate multiple antioxidant systems including GSH and thioredoxin, buffering ROS levels to allow tumor cell progression (Harris et al., 2015). Although thioredoxin is not so abundant as GSH in cells, it reduces ROS and is regenerated in a GSH-independent manner by thioredoxin reductase (Holmgren and Lu, 2010). Because GSH and thioredoxin pathways synergistically contribute to cancer cell survival, it has been suggested that blocking both GSH and thioredoxin pathways inhibits cancer promotion (Harris et al., 2015). Cancer cells show metabolic alterations to manage oxidative stress, and therefore, a recent study has suggested that glutathione synthetic pathway is a promising therapeutic target (Beatty, 2015). Mass spectrometry was used to conduct metabolomics profiling of triple-negative breast cancer (TNBC) compared with control cells (Beatty, 2015). TNBC does not represent oncogenic HER2 amplification. It does not express estrogen receptor or progesterone receptor. TNBC is an aggressive and genetically heterogeneous subset of breast cancer, which is refractory to usual targeted therapies (Beatty, 2015). A distinct feature of TNBC metabolic profiling is that levels of glutathione, a cellular redox buffer, are lower in TNBC cell lines compared with the controls (Beatty, 2015). Glutathione biosynthesis is required to suppress ROS in TNBC cells. Thus, inhibition of glutathione biosynthesis leads to reduced tumor cell growth both *in vitro* and *in vivo* (Beatty, 2015), illustrating the role of GSH metabolic alterations in cancer. Metabolomics contributes to a better understanding of cancer therapeutically. Likewise, malignant mesothelioma is a fatal cancer with no effective cure. Recently, disabling mitochondrial peroxide metabolism or reducing Akt signaling suppressed mesothelioma malignancy (Tomasetti et al., 2014; Cunniff et al., 2015). This finding may be linked to the examination showing that ROS induced by high dose of ascorbate in mesothelioma inhibited cell death (Takemura et al., 2010).

## Effect of Vitamin C on GSH Metabolism and Glucose Metabolism

Vitamin C (L-ascorbic acid) is a well-known reducing agent that is easily oxidized to dehydroascorbate (DHA) in solution. Physiologically, vitamin C is transported into cells as ascorbate in specific cell types by sodium-dependent ascorbic acid transporters. It can also be administered into cells in oxidized DHA form facilitated by glucose transporters (GLUTs) (Nishikimi and Yagi, 1991; Vera et al., 1993; Tsukaguchi et al., 1999; Rumsey et al., 2000; Liang et al., 2001). Following the transportation of DHA into cells via glucose transporters, it is reduced to ascorbate using GSH, and is trapped inside the cells where it accumulates as ascorbic acid (Vera et al., 1993, 1995). Therefore, vitamin C is considered as a pro-oxidant that produces oxidative stress (Halliwell and Foyer, 1976; Heikkila and Cabbat, 1983). Accordingly, vitamin C enhances arsenic trioxide (As_2_O_3_)-induced cytotoxicity in multiple myeloma cells by decreasing intracellular GSH levels (Grad et al., 2001). A clinical study has reported such results in patients with multiple myeloma treated with a combination of vitamin C and As_2_O_3_ (Bahlis et al., 2001). *In vitro*, vitamin C suppresses the growth of mouse myeloma cells. *In vivo*, vitamin C inhibited the growth of leukemic progenitor cells isolated from a patient with acute myeloid leukemia (AML) in our previous study (Park et al., 1971, 1992; Park, 1985). In a few clinical studies, manipulation of vitamin C levels in AML patients has produced clinical benefit (Park et al., 2001, 2002). Based on such result, complementary and alternative medicine practitioners have used high concentrations of vitamin C to treat their patients (Meister and Anderson, 1983; Park et al., 2001, 2002; Park, 2013). The physiological concentration of vitamin C is <0.1 mM in plasma. Plasma vitamin C concentrations (1–10 mM, depending on cell lines) that are toxic to cancer cells *in vitro* can be attained clinically by i.v., and not via oral administration of a high dose of vitamin C (Park, 2013).

Recent studies have found that serum concentrations of GSH are associated with various disease conditions (Droge and Breitkreutz, 2000; Prousky, 2008; Forman et al., 2009; Smeyne and Smeyne, 2013). For example, decreased serum concentration of GSH has been linked to cancer and neurodegenerative disease susceptibility (Smeyne and Smeyne, 2013). Because GSH is so poorly absorbed in the gastrointestinal system, i.v. GSH (rather than most oral GSH supplements) represents another complementary and alternative medicine therapy (Chen et al., 2011).

We have previously reported that *in vitro* treatment with 0.25–2.0 mM vitamin C induces apoptosis of leukemia cells (Park et al., 2004). Vitamin C-stimulated oxidation of GSH to dimerized oxidized form (GSSG) leads to accumulation of hydrogen peroxide (H_2_O_2_), resulting in the induction of apoptosis. A number of previous reports also suggested that high-dose vitamin C kills cancer cells by acting as a pro-drug that generates H_2_O_2_ (Chen et al., 2008; Takemura et al., 2010; Du et al., 2012; Uetaki et al., 2015). The direct role of H_2_O_2_ in the induction of apoptosis in acute myeloid leukemia (AML) cells has been confirmed using catalase to completely abrogate vitamin C-induced apoptosis (Park et al., 2004).

A recent metabolomics study has suggested an important relationship between vitamin C and GSH in terms of glucose metabolism, including glycolysis, citric acid cycle (tricarboxylic acid; TCA cycle), and pentose phosphate pathway (Uetaki et al., 2015). A list of metabolites associated with metabolic perturbations related to glucose metabolism is provided in **Table 2**, which is in line with a previous report showing that vitamin C influenced glucose metabolism (Hwang et al., 2009; Yun et al., 2015). Levels of metabolites associated with upstream glycolysis, partial TCA cycle (such as citrate and cis-aconitate), and pentose phosphate pathway (PPP) are increased in response to high-dose vitamin C while levels of metabolites downstream of glycolysis are decreased with the exception of citrate and cis-aconitate (Uetaki et al., 2015). This finding may be attributed to GAPDH inactivation by vitamin C-induced oxidative stress. Upstream metabolites of GAPDH accumulate whereas downstream metabolites of GAPDH were depleted. ATP concentrations are decreased in response to high-dose vitamin C, indicating that high-dose vitamin C interfered with glycolytic and citric acid cycle energy flux, consequently diminishing ATP production (Uetaki et al., 2015). The authors have suggested that vitamin C-mediated oxidative stress in turn induces the depletion of NADH, which inhibits glycolytic flux. The decreased ATP level due to inhibition of energy metabolism causes cell death (Uetaki et al., 2015).

**Table 2:** Metabolites of glucose and GSH metabolism altered by vitamin C (Metabolite alteration by high concentration (>1 mM) of vitamin C in glucose and GSH metabolism).

Metabolite	Regulation	Cancer type (Reference)
Glucose 6-phosphate	Up	MCF7 breast cancer (Uetaki et al., 2015). Hepatocellular carcinoma, Serum (Gao et al., 2015). Primary ovarian cancer (Gao et al., 2015)
Fructose 6-phosphate	Up	MCF7 (Uetaki et al., 2015)
Fructose 1,6-bisphosphate	Up	MCF7 (Uetaki et al., 2015)
Dihydroxyacetone phosphate	Up	MCF7 (Uetaki et al., 2015)
3-Phosphoglycerate	Down	MCF7 (Uetaki et al., 2015)
Phosphoenolpyruvate	Down	MCF7 (Uetaki et al., 2015)
Pyruvate	Down	MCF7 (Uetaki et al., 2015)
Lactate	Down	MCF7 (Uetaki et al., 2015)
Citrate	Up	MCF7 (Uetaki et al., 2015)
Isocitrate	Down	MCF7 (Uetaki et al., 2015)
α-Ketoglutarate	Down	MCF7 (Uetaki et al., 2015)
Fumarate	Down	MCF7 (Uetaki et al., 2015)
Malate	Down	MCF7 (Uetaki et al., 2015)
ATP	Down	MCF7 (Uetaki et al., 2015). K-Ras, B-Raf colorectal cancer (Yun et al., 2015). SH-SY5Y neuroblastoma (Ma et al., 2017)
ADP	Up	MCF7 (Uetaki et al., 2015)
AMP	Up	MCF7 (Uetaki et al., 2015)
GTP	Down	MCF7 (Uetaki et al., 2015)
GDP	Up	MCF7 (Uetaki et al., 2015)
GSH	Down	MCF7 (Uetaki et al., 2015). HepG2 (Wang et al., 2016). Non-small-cell lung cancer (Brunelli et al., 2014)
GSSG	Up	MCF7 (Uetaki et al., 2015). Non-small-cell lung cancer (Brunelli et al., 2014). Breast cancer (Willmann et al., 2016). Oral cancer (Ishikawa et al., 2016). Breast cancer (Tang et al., 2014)
Cys-containing protein/peptide	Up (within 1 h)	Leukemia (Park, 2007)

GSH plays a significant role in cellular defense against oxidative stress by reducing free radicals and ROS. It acts in various cysteine-mediated intracellular processes, including the metabolism of cysteine amino acids and biosynthesis of leukotrienes and DNA (Larsson et al., 1983; Meister and Anderson, 1983). GSH is synthesized via sequential steps of two enzyme reactions containing γ-glutamylcysteine synthetase (γ-GCS) and GSH synthase. γ-GCS catalyzes the rate-limiting step of GSH synthesis (Meister and Anderson, 1983). GSTs are a major group of detoxification enzymes that conjugate GSH to reactive metabolites. Multiple forms of GST isozymes have been identified (Shepherd et al., 2000). To date, eight distinct classes (α, κ, μ, φ, π, θ, σ, and ζ) encoding soluble cytosolic GSTs have been identified in mammals on the basis of their degree of sequence identity (Hayes and McLellan, 1999). GST-P1 is a gene that encodes a GST belonging to the π class. GST-A1, A2, A3, and A4 genesc encode human GST subunits belonging to the α class. GST-M1, M2, M3, M4, and M5 genes encode GST subunits belonging to the μ class (Sheehan et al., 2001). Substantial evidence suggests that ROS play an important role in cellular signaling linked to transcriptional machinery or act as a second messenger (Griffith and Meister, 1979; Palmer and Paulson, 1997; Kunsch and Medford, 1999; Hensley et al., 2000; Sheehan et al., 2001; Carcamo et al., 2002a,b). Furthermore, evidence indicates that phase II detoxification enzymes such as GSH S-transferase, NAD(P)H:quinone oxidoreductase1, UDP-glucuronosyltransferase, and epoxide hydrolase can be induced by various compounds, including food phytochemicals (Wattenberg, 1981; Nakamura et al., 2000). Our previous data established the regulation of GSH levels via transcriptional regulation of glutathione synthase and GST synthesis by vitamin C (Park, 2007). The role of vitamin C-induced changes in GSH/GSSG ratio was first established in this report. We have investigated the relationship of vitamin C with GSH in leukemia cell lines. We found that vitamin C-induced decrease in intracellular GSH/GSSG ratio and H_2_O_2_ accumulation led to transcriptional induction of intracellular protein and protection against oxidative stress, such as γ-GCS in HL-60 and NB-4 cells. Although the effect of H_2_O_2_ accumulation induced by vitamin C was eliminated by catalase, vitamin C-mediated transcriptional induction of these enzymes has been observed, indicating that the altered GSH/GSSG ratio was more important than H_2_O_2_ accumulation in inducing the activity of enzymes that protect against oxidative stress (Park, 2007).

A redox cycle requires adequate support via GSH reductase and GSH peroxidase for defense against redox stress. In addition, relatively high concentrations of GSH via synthesis and active transport of GSSG or GSH S-conjugates are needed. Stimulation of γ-GCS transcription increases GSH concentration (Goto et al., 1995). Our observations suggested that vitamin C stimulated the expression of γ-GCS, resulting in an increase in the level of GSH *via de novo* synthesis at the expense of cysteine (Park, 2007). Concentrations of GSH in three types of myeloid leukemia cells were elevated within 3 h after treatment with vitamin C and gradually returned to their baseline levels by 12 h (Park, 2007). Such increase in the concentration of GSH was associated with enhanced expression of γ-GCS. GSH synthesis and GST activation in response to vitamin C occurred rapidly (in 1 h) (Park, 2007). The elevated expression of γ-GCS in response to vitamin C is accompanied by corresponding increase in the concentration of GSH, representing an important function of vitamin C in cellular GSH homeostasis (Park, 2007).

Cysteine is known to be a rate-limiting precursor for GSH synthesis (Watanabe and Bannai, 1987). Therefore, we investigated cysteine uptake in AML cells after treatment with vitamin C (Park, 2007). Intracellular L-Cys incorporation was measured in intact HL-60, NB4, and KG1 cells exposed to vitamin C using ^35^S-labeled-L-Cys containing media (Park, 2007). The rate of uptake in the absence of vitamin C was very low (at most 119% of baseline by 16 h) (Park, 2007). However, it peaked after 1 h and 3 h (Park, 2007). An inhibitor of gamma-glutamylcysteine synthetase, buthionine sulfoximine potently inhibited the second peak, suggesting glutathione synthesis following the incorporation of cysteine. These results indicate that vitamin C induced GSH synthesis in parallel with intracellular cysteine uptake. Interestingly, intracellular GSH levels in these AML cells incubated with vitamin C peaked around 3 h and declined thereafter, while the increase in [^35^S]-L-Cys incorporation occurred at 3 h and continued (Park, 2007). This result demonstrated that transporation of [^35^S]-L-Cys into cells through cysteine uptake is followed by incorporation and intracellular transfer. Thus, the sulfhydryl transfer system might be affected by vitamin C.

In view of the signaling effects of vitamin C, the association between vitamin C and glutathione in myeloid cells may partly explain the potential effect of vitamin C on cellular signal transduction. It appears that vitamin C has a positive effect on sulfhydryl (-SH) uptake. Considering that intracellular concentration of glutathione determines cellular thiol-disulfide redox potential to a large extent, it might regulate a variety of cellular processes via disulfide bridge formation and protein glutathionylation.

## Conclusion

**FIGURE 1:**
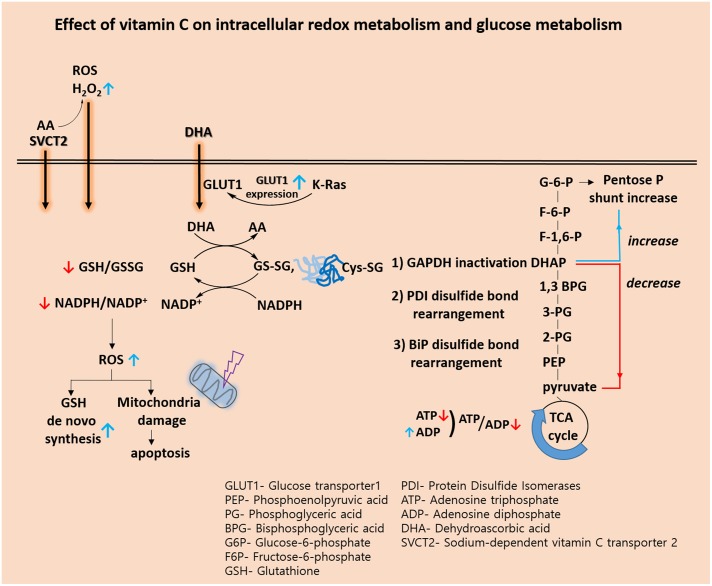
Effect of vitamin C on intracellular redox metabolism and glucose metabolism.

Recently, biological and pre-clinical studies suggest that high dose intravenous vitamin C combined with conventional chemotherapy agent synergistically increase the effectiveness of cancer therapy. (Espey et al., 2011; Hoffer et al., 2015). A phase I study states that high dose intravenous vitamin C in combination with gemcitabine and erlotinib in patients with metastatic pancreatic cancer did not reveal increased toxicity (Monti et al., 2012). In view of the metabolic effect, we conclude that vitamin C plays a key role in the challenges associated with glucose and GSH metabolism (**Figure 1** and **Table 2**). Vitamin C induces high ROS level and oxidation of GSH. Accompanied by direct glutathionylation of GAPDH in glycolysis, glucose metabolism was altered by vitamin C treatment. Furthermore, changes in reduced glutathione ratio triggered by vitamin C resulted in altered GSH metabolism via *de novo* synthesis. From the information available, it seems clear that vitamin C is involved in a variety of oxidative mechanisms. Therefore, vitamin C may be an adjuvant medicine combined with conventional chemotherapy drug to induce cancer cell death. In the future, another issue pertaining to vitamin C is whether its use as an adjuvant medicine is valid in all populations or only in some populations depending on the range of intakes. Therefore, further studies are required to identify the molecular targets of vitamin C sensitivity such as transporter.

## Author Contributions

SP conceived the idea and wrote the original draft. SA and YS drafted the manuscript. YY and CY reviewed and supervised the manuscript writing process.

## Conflict of Interest Statement

The authors declare that the research was conducted in the absence of any commercial or financial relationships that could be construed as a potential conflict of interest.
